# The Oslo Health Study: A Dietary Index Estimating Frequent Intake of Soft Drinks and Rare Intake of Fruit and Vegetables Is Negatively Associated with Bone Mineral Density

**DOI:** 10.4061/2011/102686

**Published:** 2011-07-02

**Authors:** Arne Torbjørn Høstmark, Anne Johanne Søgaard, Kari Alvær, Haakon E. Meyer

**Affiliations:** ^1^Section of Preventive Medicine and Epidemiology, University of Oslo, P.O. Box 1130, Blindern, 0318 Oslo, Norway; ^2^Division of Epidemiology, Norwegian Institute of Public Health, P.O. Box 4404, Nydalen, 0403 Oslo, Norway

## Abstract

*Background*. Since nutritional factors may affect bone mineral density (BMD), we have investigated whether BMD is associated with an index estimating the intake of soft drinks, fruits, and vegetables. *Methods*. BMD was measured in distal forearm in a subsample of the population-based Oslo Health Study. 2126 subjects had both valid BMD measurements and answered all the questions required for calculating a *Dietary Index* = the sum of intake estimates of colas and non-cola beverages divided by the sum of intake estimates of fruits and vegetables. We did linear regression analyses to study whether the Dietary Index and the single food items included in the index were associated with BMD. *Results*. There was a consistent negative association between the Dietary Index and forearm BMD. Among the single index components, colas and non-cola soft drinks were negatively associated with BMD. The negative association between the Dietary Index and BMD prevailed after adjusting for gender, age, and body mass index, length of education, smoking, alcohol intake, and physical activity. *Conclusion*. An index reflecting frequent intake of soft drinks and rare intake of fruit and vegetables was inversely related to distal forearm bone mineral density.

## 1. Introduction

It is generally agreed that nutritional factors are important for the development of osteoporosis. Among several negative factors for bone formation are sodium, protein, caffeine, oxalate, fibre, phytate, and increased acid load, whereas calcium, vitamin D, salads, herbs, and vegetables seem to be bone promoting factors. Also alkali buffers, whether bicarbonate, vegetables, or fruits, can reverse the urinary calcium loss [1–4]. 

During the last decades consumption of soft drinks has increased in most Western countries. The consequences on bone health of this consumption have been relatively sparsely investigated. Some studies have reported an inverse association between consumption of cola beverages/soft drinks and bone health [5, 6], and other studies found the same association only in females [7–10]. Finally, one study in older women concluded that moderate intake of carbonated beverages did not appear to have any adverse effects on bone mineral density [11]. Thus, there are inconsistent results concerning the possible influence of soft drinks on bone health. 

In the present work, we have examined whether data of the Oslo Health Study would fit the hypothesis that intake of some diet items would reduce, whilst intake of others would improve the bone mineral density. As potential negative diet items for bone health we have focused on soft drinks (colas and non-cola soft drinks); fruits and vegetables were chosen as positive diet factors [1]. We reasoned that a negative dietary influence on BMD might be more easily detected by a Dietary* Index* calculated as the sum of intake estimates of colas and non-cola beverages divided by the sum of intake estimates of fruits and vegetables. Therefore, the aim of this work was to study the association between BMD and both the Dietary Index and its single components.

## 2. Materials/Subjects and Methods

### 2.1. Main Project

The Oslo Health Study (HUBRO) was carried out in 2000-2001 under the joint collaboration of the Norwegian Institute of Public Health, the University of Oslo, and the Municipality of Oslo. All residents of Oslo born in 1970, 1960, 1955, 1940-41, and 1924-25 were invited to the survey station, and 18 770 participated (42% in men and 49% in women). The participation rate in the different age groups is presented previously [12]. HUBRO comprised a simple physical examination including measuring of height, weight, waist and hip circumference, blood pressure and heart rate, and collection of nonfasting venous blood analyzed for serum lipids and glucose. One self-administered questionnaire was part of the letter of invitation, whereas two supplementary questionnaires were handed out at the screening station and returned in pre-stamped self-addressed envelope. The questionnaires provided information on health status, symptoms, diseases, and various aspects of health behaviour—including questions about intake of food and drinks. Because subjects aged 75-76 years were not asked about cola intake, they were not included in this study.

Up to two reminders were sent to the nonresponders of the survey. All procedures at the screening station were performed by experienced and trained personal following a detailed protocol. More detailed information could be found at HUBRO's web-site: http://www.fhi.no/hubro-en.

### 2.2. Ethics

The study protocol was placed before the Regional Committee for Medical Research Ethics and approved by the Norwegian Data Inspectorate. The study has been conducted in full accordance with the World Medical Association Declaration of Helsinki.

### 2.3. The Osteoporosis Substudy

As part of HUBRO, all women born in 1941 (59 years of age) who had participated in a health survey in 1981 and a random sample of the other age and gender groups were selected to an Osteoporosis substudy and offered measurement of bone mineral density (BMD) of the nondominant forearm. All together 2690 subjects were measured which represent 37.0% of the factual number selected to meet at the screening station. For more detailed information, see [13].

### 2.4. Assessment of Bone Mineral Density (BMD)

BMD was measured by single energy X-ray absorptiometry (SXA; DTX-100, Osteometer MediTech Inc., Hawthorne, Calif, USA) at both the distal and ultradistal forearm site. The region of interest was automatically identified by the computer. The distal site included a fixed length of 24 mm of both radius and ulna, distantly starting at the point where the distance between radius and ulna is 8 mm (8 mm point). The ultradistal site included radius from the 8 mm point to the radial endplate. All the scans were reviewed by one operator, and corrections made when artefacts appeared [13]. In the present study, we have reported data only at the distal site (10–20% trabecular bone) in tables to simplify the presentation. The results of the ultradistal site are referred in the text. More details about the measurement procedures and the quality control are published before [13, 14].

### 2.5. Calculation of the Dietary Index

HUBRO has questions about diet items and beverages that potentially could influence the bone health, such as colas and non-cola soft drinks, fruit, berries, and vegetables. Questions related to protein intake were, however, hard to appreciate and were not included in the index.

The questions for intake frequency of food items had six levels of answers: 1 = rarely/never; 2 = 1–3 times per month; 3 = 1–3 times per week; 4 = 4–6 times per week; 5 = 1-2 times per day; 6 = 3 or more times per day. For beverages there were five levels: 1 = rarely/never; 2 = 1–6 glasses per week; 3 = 1 glass per day; 4 = 2-3 glasses per day; 5 = 4 or more glasses per day. The food frequency questions do not allow calculation of “true” intake frequencies or amounts, and no correction was made for variations in units, that is, 6 levels for food items (from rarely/never to 3 or more times per day) and 5 levels for beverages (from rarely/never to 4 or more glasses per day). The Dietary Index was then constructed based upon the literature about whether a food item was classified as positive or negative for bone health. We used the intake frequency levels (1 to 6 for foods and 1 to 5 for beverages) as crude estimates of intake frequencies. The index was then defined as the sum of intake frequency levels of colas and non-cola soft drinks (sum of two item, range 2–10) divided by the sum of intake frequency estimates of fruit/berries, fruit juice, cooked vegetables, and raw vegetables/salads (sum of four items, range 4–23). Thus, the lowest value for the index was 0.08 and the highest value was 2.50. Thus, the rationale for calculating the dietary index was that bone mineral density is expected to be negatively related to factors in the nominator and positively to those in the denominator. It was required that all subjects of the study had values >0 for all components of the dietary index, thereby avoiding zero values for the nominator and denominator.

We also calculated an alternative index, using times per week for the intake frequencies, using midpoints of the frequency intervals. For example, intake frequency 1-2 times per day would be 1.5∗7 = 10.5 times per week, and 1–3 times per month would be 2/4 = 0.5 times per week. The response alternative “rarely/never” was set to zero (actually 0.01 to be able to calculate) times per week, and “4 glasses or more per day” was defined as 28 glasses per week. Thus, both the lowest and highest response alternatives would underestimate the true frequencies. In any instance, none of the approximations would give information of intake amounts, or even true intake frequencies. 

In the statistical analyses, we chose to include only subjects having data on all of the components of the Dietary Index. The material therefore consisted of 2126 subjects. The distribution of the Dietary Index was slightly skewed with the tail towards higher values. The mean values were in the range from 0.2 to 0.4 in the various age groups (Table 1).

### 2.6. Covariates

Body Mass Index was defined as weight in kg divided by height in meter squared and was measured without shoes and with light indoor clothing. The questionnaires provided information about the other covariates. Smoking status was categorized as daily smokers versus nonsmokers/former smokers. Years of education was used as a continuous variable. The question about alcohol consumption had 8 categories, from 4–7 times per week to never drunk alcohol. The variable was dichotomized into those drinking 1–7 times per week versus those drinking less frequent. Physical activity was measured by a question about hours per week of light physical activity during leisure time the previous year, including going to/from work. The categories were 0, <1, 1-2, 3 or more hours per week.

### 2.7. Statistical Methods

Linear regression was used to evaluate the association between intake frequency estimates of various single food items, the Dietary Index, and bone mineral density at the distal forearm. The distribution of bone mineral density (BMD) was fairly normal (not shown). We first studied the association between some single food items supposed to influence the acid load and BMD, adjusting for gender, age, body mass index, length of education, smoking, alcohol intake, and leisure time physical activity. The regression analyses were performed with one by one of the items of food/beverages as main independent variable. We then did the same analysis with all the food/beverage items entered simultaneously in the model. Each item was adjusted for all the other items and the covariates. Next, we studied the association between the *Dietary Index* and BMD, in several Models: (1) adjusting for gender and age only, (2) the preceding + body weight + body height, (3) the preceding + years at school, and (4) the preceding + smoking + alcohol + leisure time physical activity. We also used Model (4) to analyze the association between BMD and the Dietary Index in women and men separately. Finally, the number of components in the index was reduced by omitting one by one of its components in linear regression models, examining the influence of this procedure on the association between the various new indexes and BMD. 

A significance level of 0.05 was accepted. SPSS 15.0 was used for the analyses. 

## 3. Results

### 3.1. Characteristics of the Study Population

Table 1 shows the percentage or mean (SD) for the variables used in this study, by age and gender. In all age groups, the *Dietary Index* was higher for men than women, and the index decreased with increasing age.

### 3.2. Intake of Single Food Items/Beverages and Distal Forearm BMD

When we entered the food items one by one, cola and non-cola soft drinks were negatively associated with distal forearm BMD, whereas intake of raw vegetables was positively associated, when the covariates were adjusted for (Table 2). However, entered simultaneously, none of the items showed a statistically significant association with distal forearm BMD. Stratified analyses on gender gave similar results, with the exception that intake of cooked vegetables showed a negative association (*P* = .039) in men (results not shown).

### 3.3. The Dietary Index and Distal BMD

There was a significant inverse relationship between the Dietary Index and distal BMD when adjusting for gender and age (Table 3, Model (1)). Subsequently, we adjusted for an increasing number of possible covariates in models (2)–(4) (Table 3). The *β* coefficient remained almost constant when adding variables such as body mass index, years at school, smoking, alcohol intake, and leisure time physical activity. Additional adjustment for milk consumption (5 response alternatives) did not change the result (*β* coefficient = −0.050).

An additional question asked the respondents whether their consumption of soft drinks were mainly with sugar or without. Of those answering this question, 35% mainly used soft drinks without sugar. Taking this variable into the regression analyses did not change the results. 

The analyses in Table 3 were also done for BMD at ultradistal site. The unstandardized (*B*) and standardized (*β*) regression coefficients were slightly less negative, but the pattern was the same (results not shown).

### 3.4. Linear Regression Analyses in Each Gender

Model (4) in Table 3 was then used to analyze the association between BMD and the Dietary Index in women and men, separately. There was still a significant association in men (*P* = .03) and women (*P* = .05), results not shown.

### 3.5. Reducing the Number of Variables in the Dietary Index

We finally studied the influence of reducing the number of components in the Dietary Index, by omitting one by one of its components, starting with fruit juice and ending with non-cola soft drinks, leaving cola solely in the formula (Table 4). Adjusting for the covariates mentioned in Table 2, the *β* values were gradually attenuated from −0.050 to −0.040 but the association remained significant (Table 4).

## 4. Discussion

The present findings would seem in keeping with the view that intake of fruits and vegetables acts positively and soft drinks negatively on bone mineral density. However, cross-sectional data are not suitable to clarify whether associations are causal. The Dietary Index probably reflects an unhealthy lifestyle in general, and mechanisms to explain the observed associations are not clarified by the present results. Still, the consistency of our findings is noteworthy, and we will shortly discuss some possible mechanisms, based upon current literature. 

In the rat, indigestible carbohydrates of plants, such as fructo-oligo-saccarides, have been shown to increase intestinal calcium absorption, calcium balance, and bone mineral density [15, 16]. Among mechanisms of action are increased absorption area of the large bowel produced by a high fibre content and formation of short-chain fatty acids which could promote calcium absorption in the colon. Also fibre components of fruits and vegetables might have these effects. Green vegetables may also promote retaining of calcium through their vitamin K content [17] and possibly imply that frequent use of vegetables may increase BMD. 

Like coffee, also colas contain caffeine, and this methyl xanthine may be a risk factor for osteoporotic fractures [18]. It has, however, been questioned whether caffeine intake has any harmful effect on bone health in subjects ingesting adequate levels of calcium [19].

With reference to literature, many of the diet items of the Dietary Index could, in theory, influence the acid load. In general, a food may be classified as either acidic or alkaline by its influence on the urine pH. We emphasize that many of the index components provide acid when first entering the gastro intestinal system, such as colas and other types of soft drinks, fruits, and berries. Thus, a local acid effect, for example, on the teeth is expected, irrespective of type of the acid. However, their final effect on the acid load of the body could differ appreciably, depending on the metabolic fate of the ingested acids. Vegetables and fruits have been shown to produce more alkaline urine [20]. Thus, contrary to what might be expected, even fruits with a sour taste give a reduced acid load due to their high content of salts of organic acids [21]. A high intake of fruits and vegetables should accordingly lower the acid load and reduce the urinary loss of calcium. In keeping with these considerations, we previously reported an association between hip fractures and a low intake of vegetables and fruits [22]. In the present study, we observed a positive association between BMD and intake of raw vegetables, but only with borderline significance. It is tempting to suggest that the present results could be of some interest when considering prevention and treatment of osteoporosis, but more research is needed.

A high intake of sugar sweetened soft drinks may give an increased acid load partly because of the presence of carbonic acid, citric acid, and, in colas also, phosphoric acid [23, 24]. Additionally, also the fructose component of sucrose seems to have the ability to increase the acid load due to uric acid formed during catabolism of fructose in the liver [25]. As discussed by Barzel and Jowsey [26], intake of colas may increase the acid load and have a negative effect on the skeleton. Although the phosphorus content in colas is higher than in most other beverages, others have suggested that it is not high enough to yield a relevant amount of dietary acidity [27]. However, in this context it is crucial to examine the acid potential of dietary phosphate, that is, whether phosphate is monobasic, dibasic, or tribasic [3]. 

Several studies indicate that ingestion of low doses of acids can increase urinary calcium excretion in man and increase the risk of osteoporosis and bone fractures, whereas intake of alkali buffers such as bicarbonate may reduce bone loss [28–33]. However, one study found a weak association between decreased bone health and acid load only in women [34], and a few studies did not find any association at all [11, 35]. In a meta-analysis, Fenton et al. [36] concluded that there is no evidence that higher phosphate intakes are detrimental to bone health, and the same author reported in a more recent study that urine pH and urine acid excretion did not predict osteoporosis risk [37]. 

As discussed by Heanay and Layman [38], intake of protein may have both negative and positive effects on bone health depending on a variety of factors, but the impact of protein on acid production seems to be minor compared with the alkalinizing effects of fruits and vegetables. Thus, currently the literature seems to provide ambiguous information on the significance of protein intake on bone health. 

Although basic physiology and even early experimental studies suggest a dietary acid load might increase the risk of osteoporosis [26, 39], it is not clarified whether the modern Western diet might give an acid load of a magnitude which could affect the bone mineral density in man. In a Canadian prospective population-based study, the researchers found two underlying dietary patterns in the participants; one was composed of intake of fruits, vegetables, and whole grains (named “nutrient dense”), the other consisted of intake of soft drinks, fast food, certain meats, and sugar-containing desserts (named “energy dense”) [40]. In subgroups of the participants, the nutrient dense factor was associated with increased BMD when adjusted for body mass index, whereas the energy dense factor was associated with decreased BMD. They concluded that some factors related to the energy dense dietary pattern, which in their study resulted in increased BMI, may partially offset the advantages of higher body mass index with regard to bone health [40]. Possibly, one explanation of their results could be that the negative effect of the probably more acidic energy dense diet would counterbalance the positive effect of increased BMI, an interpretation which would seem in support of the present results. 

In Norway, the annual turnover of carbonated beverages increased fifteen times between 1950 and 2002, but then levelled off [41], which means that there was a high consumption in the years before collection of the present data in 2000. 

It has been suggested that negative effects of cola beverages could be attributed to the content of phosphoric acid, and two studies in rats have found that cola consumption induces reduction in bone mineral density [42, 43]. The last one suggests that the decrease in BMD might be related to the renal damage caused by cola drinks in addition to other related factors [43]. Recently, cola intake was shown to acutely increase the calcium excretion in women [44]. However, a meta-analysis concluded that there is no evidence that higher phosphate intakes, regardless of protonation of phosphate, are detrimental to bone health [36]. 

Although there has been a decrease in the consumption of sugar-sweetened soft drinks among children and adolescents during the last years in Norway, the consumption is still among the highest in the world. Almost 50% of the total consumption of sugar in Norwegian youth comes from sugar-sweetened soft drinks and lemonade [45]. The negative consequences on bone health might become significant in the long term, because of a possible lower peak bone mass. 

Using cola as an alternative to milk would be expected to reduce calcium supply as well. The data of the present study did not show any correlation between cola consumption and milk intake, and additional controlling for milk in the analyses in Table 3 did not change the results. Thus, it does not seem that intake of colas or other sweetened soft drinks in our study of adults are consumed at the expense of milk.

This study has several limitations. First, the oldest subjects are 59/60 years since those 75/76 years did not get the question about consumption of cola. Thus the individuals with the highest risk of low BMD are not included. Second, our results are based upon cross sectional data. Hence, conclusions about cause-and-effect associations would be inappropriate. Third, we may have information bias as regards self-reported intake frequencies of food items and beverages. Furthermore, there is a chance of unmeasured remaining confounding variables. The questionnaire data do not allow calculation of exact intake amounts of various foods, or even their intake frequencies using the same unit, since one response alternative was “rarely/never”. We therefore applied 1 to 6 linear levels for intakes of food items and 1 to 5 levels for the beverages. Although our estimates of food and beverage intakes are not reflecting true intake frequencies or amounts, it is likely that the classification of self reported intakes into six (or five) linear levels would serve to illustrate variations in the intake pattern between groups of subjects. Thus, we have used the units only as a crude estimate to illustrate the intake frequency pattern of various diet items. However, the BMD impacts of corresponding levels of the various diet items are not directly comparable. On the other hand, it is hard to appreciate how the units should be weighted. The alternative calculation, where intake frequencies were based upon midpoint approximations within each food category, turned out to give a less consistent picture of the association between the Dietary Index and bone mineral density.

## 5. Conclusion

The present results on distal forearm BMD could be in favour of the view that frequent use of soft drinks and rare intake of fruits and vegetables might have a negative influence on the skeleton, but the mechanisms involved are not clarified.

## Figures and Tables

**Table 1:** Characteristics of the study sample (*n* = 2126).

	Age group (years)		
	30	40 + 45	59–60
*Men*	*n* = 246^a^	*n* = 396^a^	*n* = 229^a^
Body weight (kg)	82.7 (12.4)^b^	83.5 (13.1)	82.6 (12.3)
Height (cm)	180.2 (7.5)	178.1 (11.0)	175.7 (12.6)
BMD (g/cm^2^)	0.57 (0.04)	0.56 (0.05)	0.53 (0.06)
Dietary Index^c^	0.38 (0.20)	0.32 (0.19)	0.25 (0.14)
(i) Colas	2.20 (1.05)	1.90 (1.01)	1.55 (0.92)
(ii) Non-colas	1.84 (0.74)	1.65 (0.87)	1.45 (0.73)
(iii) Fruit/berries	3.38 (1.15)	3.51 (1.22)	3.78 (1.14)
(iv) Cooked vegetables	2.81 (0.99)	3.10 (0.94)	3.51 (0.94)
(v) Raw vegetables	3.06 (0.96)	3.19 (1.02)	3.32 (1.09)
(vi) Fruit juice	2.30 (0.96)	2.10 (0.92)	2.08 (0.89)
Education (years)	15 (3)	14 (4)	13 (4)
Daily smokers (%)	26.8	34.7	27.0
Use of alcohol 1 to 7			
Times per week (%)	55.9	57.4	65.2
PA (%)^d^			
(i) No activity	6.0	9.6	8.2
(ii) <1 h/wk	12.0	14.1	11.4
(iii) 1-2 h/wk	29.6	26.6	25.4
(iv) ≥3h/wk	52.4	49.7	55.0

*Women *	*n* = 212^a^	*n* = 415^a^	*n* = 628^a^
Body weight (kg)	67.8 (13.0)^b^	68.3 (11.8)	69.9 (12.3)
Height (cm)	165.8 (16.3)	165.2 (12.6)	164.9 (8.6)
BMD (g/cm^2^)	0.46 (0.04)	0.46 (0.05)	0.43 (0.06)
Dietary Index^c^	0.28 (0.13)	0.25 (0.13)	0.21 (0.10)
(i) Colas	1.86 ( 0.86)	1.63 (0.91)	1.31 (0.69)
(ii) Non-colas	1.57 (0.66)	1.44 (0.67)	1.30 (0.58)
(iii) Fruit/berries	4.04 (1.20)	4.03 (1.19)	4.36 (1.14)
(iv) Cooked vegetables	3.03 (0.96)	3.26 (1.00)	3.59 (0.90)
(v) Raw vegetables	3.50 (0.99)	3.62 (1.04)	3.46 (1.00)
(vi) Fruit juice	2.35 (0.88)	2.06 (0.83)	1.97 (0.89)
Education (years)	16 (4)	14 (4)	12 (4)
Daily smokers (%)	22.4	32.0	23.1
Use of alcohol 1 to 7			
Times per week (%)	31.8	57.4	54.9
PA (%)^d^			
(i) No activity	3.4	7.5	4.9
(ii) < 1 h/wk	10.3	8.7	10.0
(iii) 1-2 h/wk	20.6	31.1	34.2
(iv) >3 h/wk	65.7	52.7	56.0

^a^The number may vary slightly due to missing data.

^b^Mean values, SD in parentheses.

^c^Sum of intake frequency estimates of 2 diet items (colas and non-cola soft drinks) *divided* by the sum of intake estimates of 4 items (fruit/berries, cooked vegetables, raw vegetables/salads, and fruit juice). There were 5 response alternatives for beverages and 6 for the food items (see Section 2); mean intake estimate of each index component was calculated as the mean of all levels, neglecting the variations in unit (rarely/never, times per month, week or day; see Section 2.

^d^Leisure time physical activity, % of subjects.

BMD = distal forearm bone mineral density (g/cm^2^).

**Table 2:** Association between single items of foods/beverages possibly influencing the acid load and distal forearm bone mineral density, adjusted for possible confounding variables. Linear regression.

	Adjusted for covariates^a,b^		Adjusted for all the other food items listed and the covariates^a^	
	*B* (SE)	*P*	*B* (SE)	*P*
Colas^c^	−0.003 (0.001)	.012	−0.002 (0.001)	.067
Non-cola soft drinks^c^	−0.004 (0.002)	.026	−0.003 (0.002)	.128
Fruit/berries^d^	0.001 (0.001)	.216	0.000 (0.001)	.812
Cooked vegetables^d^	0.000 (0.001)	.933	−0.001 (0.001)	.670
Raw vegetables^d^	0.002 (0.001)	.046	0.002 (0.001)	.174
Fruit juice^d^	0.001 (0.001)	.315	0.001 (0.001)	.386

^a^Gender, age, weight, height, years at school, smoking, frequency of alcohol intake, and leisure time physical activity.

^b^Performed with one by one of the items of food/beverages as main independent

^c^Five intake frequency estimates (see Section 2)

^d^Six intake frequency estimates (see Section 2).

Unstandardized (*B*) regression coefficients are shown.

**Table 3:** Four different linear regression models of the association between the Dietary Index^a^ and distal bone mineral density, as influenced by increasing number of possible confounding variables.

	*B *(SE)	*β*	*t*	*P*
Model (1) (adjusted for gender and age group)	−0.024 (0.007)	−0.050	−3.238	<.001
Model (2) (+ weight + height)	−0.026 (0.007)	−0.054	−3.490	<.001
Model (3) (+ years at school)	−0.024 (0.008)	−0.052	−3.210	.001
Model (4) (+ smoking^b^ + alcohol^c^. leisure time physical activity)^d^	−0.023 (0.008)	−0.050	−3.003	.003

^a^The Dietary Index is the sum of intake frequency estimates of 2 diet items (colas and non-cola soft drinks) *divided* by the sum of intake estimates of 4 items (fruit/berries, cooked vegetables, raw vegetables/salads, and fruit juice). There were 5 response alternatives for beverages and 6 for the food items (see Section 2).

^b^never smoker versus current or previous.

^c^1–7 times per week versus less frequent.

^d^Leisure time physical activity 0, <1, 1-2, 3 or more hours per week.

Unstandardized (*B*) and standardized (*β*) regression coefficients are shown.

**Table 4:** Association between various indexes assumed to partially reflect the acid load and distal forearm bone mineral density. The index was reduced by omitting one by one of its components: linear regression.

Index type	*B* (SE)	*β*	*T*	*P*
(1) The complete Dietary Index^a^	−0.023 (0.008)	−0.050	−3.003	.003
(2) Fruit juice	−0.017 (0.006)	−0.048	−2.869	.004
(3) Also raw vegetables	−0.010 (0.004)	−0.042	−2.452	.011
(4) Also cooked vegetables	−0.004 (0.002)	−0.044	−2.726	.006
(5) Also fruit/berries	−0.003 (0.001)	−0.048	−2.995	.003
(6) Also non-cola soft drinks	−0.003 (0.001)	−0.040	−2.523	.012

^a^The Dietary Index is the sum of intake frequency estimates of 2 diet items (colas and non-cola soft drinks) *divided* by the sum of intake estimates of 4 items (fruit/berries, cooked vegetables, raw vegetables/salads, and fruit juice). There were 5 response alternatives for beverages and 6 for the food items (see Section 2). Covariates: those mentioned in Table 2. Unstandardized (*B*) and standardized (*β*) regression coefficients are shown.
